# Hyperspectral Imaging for Identifying Foreign Objects on Pork Belly

**DOI:** 10.3390/s25227015

**Published:** 2025-11-17

**Authors:** Gabriela Ghimpeteanu, Hayat Rajani, Josep Quintana, Rafael Garcia

**Affiliations:** 1 Coronis Computing SL, 17003 Girona, Spain; gabriela.ghimpeteanu@coronis.es (G.G.); josep.quintana@coronis.es (J.Q.); 2 Computer Vision and Robotics Research Institute, University of Girona, 17003 Girona, Spain; rafael.garcia@udg.edu

**Keywords:** hyperspectral imaging, machine learning, food-processing industry, foreign body detection, vision transformer, image segmentation

## Abstract

Ensuring food safety and quality is critical in the food-processing industry, where the detection of contaminants remains a persistent challenge. This study assesses the feasibility of hyperspectral imaging (HSI) for detecting foreign objects on pork belly meat. A Specim FX17 hyperspectral camera was used to capture data across various bands in the near-infrared spectrum (900–1700 nm), enabling identification of contaminants that are often missed by traditional visual inspection methods. The proposed solution combines a segmentation approach based on a lightweight Vision Transformer with specific pre- and post-processing strategies to distinguish contaminants from meat, fat, and conveyor belt, while emphasizing on a low false-positive rate. On a test set of 55 images with contaminants, the method retained most true positives; on 183 clean images, the full pipeline eliminated all false positives. Across 208 additional images acquired under production-line temperature variation (10–55 °C), only one image exhibited small false positives, and on a challenging 95-image set with fat-like spectra the pipeline produced zero false positives. These results demonstrate high detection accuracy and training efficiency while addressing issues such as noise, temperature drift, and spectral similarity. The findings support the feasibility of real-time HSI for automated quality control.

## 1. Introduction

Computer vision solutions have been widely used in the food-processing industry to address quality and safety challenges. These systems offer economic and safety advantages over manual inspection and, in many cases, yield more consistent results. They have been applied to a number of industrial vision systems for tasks such as sorting, packaging inspection, and quality assessment of meat, fish, dairy, and produce [1]. Food safety is a critical aspect of quality control in factories, leading to the development of several non-destructive automation techniques. These methods employ hyperspectral and multispectral imaging, metal detection, X-rays, acoustic emission, ultrasound, thermal imaging, fluorescence spectroscopy, radar and terahertz (THz) imaging or near-infrared (NIR) spectroscopy [2]. However, each of these technologies has certain limitations. For example, metal detectors only detect metallic objects and struggle with small fragments embedded in meat. X-ray systems have difficulty with low-density materials such as plastics and wood and involve ionizing radiation. Infrared and NIR systems suffer from limited penetration or deliver only averaged spectral responses. Thermal imaging is affected by temperature variation, fluorescence requires fluorescent compounds, and THz imaging remains slow, noise-sensitive, and expensive [3].

Among these non-destructive methods, however, hyperspectral imaging (HSI) has emerged as a strong candidate for solving diverse problems across domains such as food safety, agriculture, environmental monitoring and medical diagnosis [4]. Within food safety, HSI has been used for applications such as detecting fungal contamination in peaches [5], quality assessment of fruits [6,7,8,9], grading of nutritional and physiochemical qualities in meat and fish [10,11], estimating egg freshness [12], and identifying adulteration of minced beef with horse meat [13]. Moreover, specifically in the context of foreign material detection, HSI has also demonstrated notable success. A 2021 study [14] combined visible near-infrared (VNIR, 400–1000 nm) and short-wave infrared (SWIR, 1000–2500 nm) hyperspectral imaging to detect foreign materials on poultry meat. Their results indicated near-100% detection accuracy for larger contaminants and up to 95% accuracy for polymers, wood, and metals at smaller sizes. Complementarily, a semi-supervised deep learning method [15] using near-infrared HSI (1000–1700 nm) for poultry meat inspection employed a generative adversarial network with a 1D U-Net discriminator, achieving 100% precision and a recall of over 93% across 30 common types of contaminants in processing plants. Another study [16] on foreign material detection on chicken meat using HSI focused on plastic contaminants. The authors paired a YOLO model for precise localization with a 1D CNN for classification of 12 common polymer types, achieving 99% detection and 97% classification accuracy. Addressing the fast-paced nature of poultry processing environments, a recent study [17] employed post-training quantization and hardware acceleration techniques to optimize inference for HSI-based foreign material detection, showcasing deployability in industrial settings. On the other hand, PA2E [18] framed the task of contaminant detection as anomaly detection using an optimized autoencoder design, outperforming the state of the art by a significant margin. These capabilities make HSI particularly well-suited for our use case.

**Figure 1:**
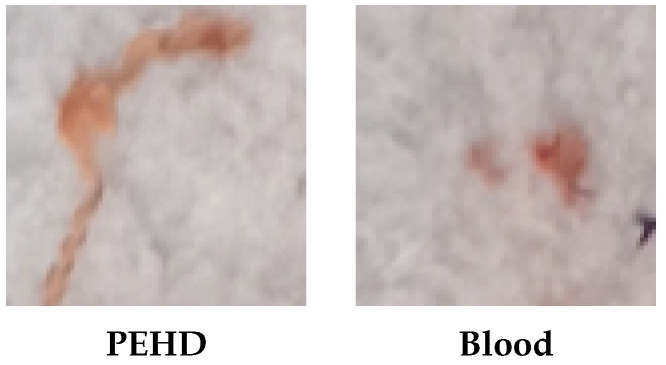
Conventional colour images highlighting the similarities between a piece of PEHD and a blood stain on pork belly.

Furthermore, in meat processing lines, products can be contaminated with foreign bodies during transportation between the slaughterhouse and packing area, often due to equipment malfunctions or human negligence. These contaminants, which may be as small as a couple of millimeters thin, pose a significant food safety risk as they frequently go unnoticed during human inspection. While conventional colour cameras are commonly employed in industrial computer vision systems, they struggle to detect such contaminants because of the similar appearance of certain materials. For instance, polyethylene high-density (PEHD) plastics and blood stains on fat can appear nearly identical, as depicted in Figure 1, while materials such as Teflon or paper can closely resemble fat. In contrast, HSI acquires both spatial information and rich spectral information across many narrow wavelengths, allowing materials with similar visual appearance to be separated based on their spectral signatures.

However, despite these advantages, hyperspectral imaging generates abundant data, creating certain challenges for computer vision tasks. The large number of internal parameters and features associated with the hyperspectral cube lead to the “curse of dimensionality” problem [19]. Moreover, in the context of machine learning, insufficient training samples can easily result in overfitting. Another critical challenge lies in the high interclass similarity and high intraclass variability in hyperspectral, necessitating sophisticated methods to achieve robust performance.

**Figure 2:**
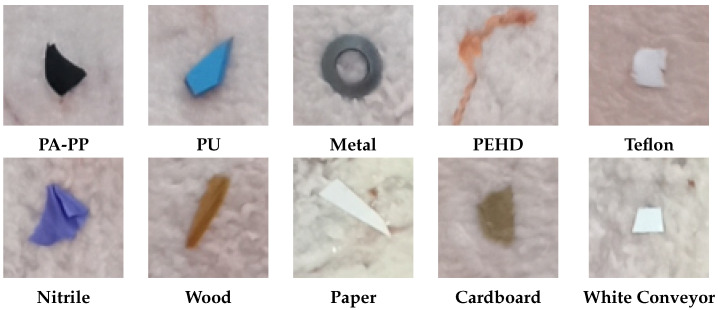
Conventional colour images depicting the ten classes of contaminants used for this study: PA-PP (polyamide (PA) plastics and polypropylene (PP) plastics were grouped into a single class due to the similarity in their spectral signatures), polyurethane (PU) plastics, metal, polyethylene high-density (PEHD) plastics, Teflon, nitrile, wood, paper, cardboard, and white conveyor belt.

In this study, we assess the feasibility of hyperspectral imaging for detecting foreign bodies on pork belly, with an emphasis on minimizing false positives and enabling real-time deployment under realistic factory settings. We formulate the problem as end-to-end semantic segmentation (or pixel-wise classification) of hyperspectral images of pork belly cuts moving along a conveyor belt. The system classifies three materials—meat, fat, and conveyor belt—as negative classes, while all other materials are considered positive detections. Potential contaminants were characterized into ten distinct classes, as illustrated in Figure 2. The contaminants included in this study were proposed by our industrial partner based on materials identified during human quality control processes and through client complaints received during routine operations. These contaminants, therefore, represent real-world foreign objects commonly encountered across multiple stages of meat processing workflows, such as slaughterhouses, boning halls, and retail preparation rooms. For example, PA fragments typically arise from wear of guide rails and gears in deboners and slicers; PP debris originates from packaging items such as tray inserts and straps; PU residue can be shed from roller coverings, knife handles, and elastomeric scrapers used for product handling and cleaning; Nitrile contaminants are primarily linked to protective equipment such as gloves; wood splinters originate from pallets and crate slats; paper and cardboard fibres come from packaging material and labels; Teflon flakes can result from degradation of non-stick coatings on heat-seal bars and chutes; and metal fragments are most often shed from blades, hooks, and fasteners in cutting, trimming, and conveying equipment, as well as from tool wear during maintenance activities. All these materials were selected in accordance with regulatory frameworks designed to ensure safety, compliance, and controlled usage within the meat processing industry.

Our main contributions can be summarized as below:

- A lightweight, end-to-end segmentation approach using a customised Vision Transformer (ViT) for detection of foreign bodies on pork belly in HSI images, improving data efficiency and reducing training time.
- A pre-processing pipeline that stabilizes performance against sensor temperature drift and exposure variation.
- A post-processing strategy that combines morphological operations with rules based on material-specific spectral characteristics, together with a tailored loss function, to reduce false positives.
- Public release of a dataset, open-source code, and pre-trained models to support further research, reproducibility, and benchmarking.

## 2. Materials and Methods

This section describes further details of the proposed pipeline. We begin with a brief summary of prior work on HSI classification. We then outline the image acquisition setup and the dataset creation procedure. Next, we explain the pre- and post-processing strategies, together with the segmentation model for foreign body detection on pork belly.

### 2.1. Related Work

Early work on hyperspectral classification focused on traditional machine learning techniques such as support vector machines (SVM) [20], *k*-nearest neighbour (*k*-NN) [21], and multilayer perceptrons (MLPs) [22]. However, the latest advancements in deep learning have introduced more powerful methods for hyperspectral image classification. Deep learning models such as Stacked Autoencoders (SAEs), Recurrent Neural Networks (RNNs) and Deep Belief Networks (DBNs) have demonstrated notable performance. Hang et al. [23] developed a model with two RNN layers that extract complementary information from non-adjacent spectral bands. While RNN-based models can effectively extract spectral features by treating the spectral signature as a sequence, they often encounter issues with vanishing gradients and struggle to learn long-range dependencies.

Due to their robust ability to capture local information, Convolutional Neural Networks (CNNs) are extremely efficient in extracting informative features from hyperspectral data. Numerous CNN-based models have thus emerged. Hu et al. [24] proposed a model for HSI classification using several 1D-CNNs that only consider spectral features. However, combining spectral and spatial features yields better performance, as demonstrated by the 3D-CNN model [25] and the 2D-CNN model [26]. HybridSN [27] employs a spectral-spatial 3D-CNN followed by a spatial 2D-CNN to model more abstract spatial representations while having a lower complexity compared to a standalone 3D-CNN. Another noteworthy model, SpectralNET [28], employs a CNN wavelet to explore both spatial-spectral domains while being computationally less expensive than a 3D-CNN. However, CNNs typically use small convolution kernels, which are effective for capturing local features but struggle to model global dependencies.

Recent works have sought to address this limitation in the context of hyperspectral image classification. Dang et al. [29] introduced a Double-Branch Feature Fusion Transformer (DBFFT) model, which uses two branches to independently extract spectral and spatial features that are then integrated by a feature fusion layer. Similarly, Roy et al. [30] proposed an attention-based adaptive spectral-spatial kernel with ResNet backbone. This approach employs spectral attention and adaptive kernel sizes, overcoming the trade-off between small kernels, which capture fine structures but miss coarse details, and large kernels, which capture coarse structures but miss finer details. Zhu et al. [31], on the other hand, proposed the spectral-spatial dependent global learning (SSDGL) framework to address challenges arising from insufficient and imbalanced HSI data. The framework incorporates a global convolutional long short-term memory module (GCL) and a global joint attention mechanism (GJAM) to improve classification by leveraging both spectral and spatial dependencies.

### 2.2. Image Acquisition Setup

**Figure 3:**
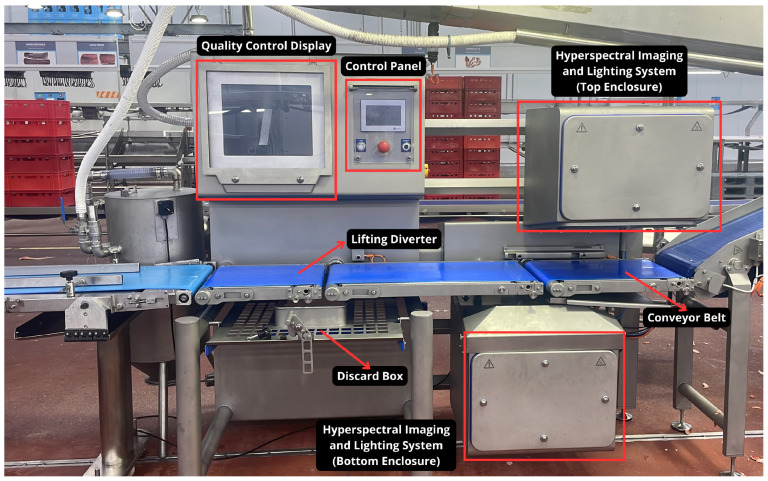
Hyperspectral image acquisition and classification setup in production.

The pork belly pieces are transported between the slaughterhouse and the packing area on a conveyor belt. To identify potential contaminants that may have entered during this process, we set up a hyperspectral camera, together with a halogen lighting system, above the conveyor belt to capture images of the pork belly pieces as they move along the belt. Figure 3 depicts this setup in production.

The hyperspectral camera used in this study was a Specim FX17 produced by Specim, Spectral Imaging Ltd. (Oulu, Finland). The Specim FX17 is a line-scan camera that collects hyperspectral data in the NIR range–a part of the spectrum not visible to the human eye and inaccessible to standard 3-channel cameras–commonly used in computer vision applications. Imaging in NIR wavelengths allows for the physical and chemical properties of materials to be determined by measuring the proportion of reflected light. Much like a person discerning the strength of tea based on its colour intensity, NIR sensors can help differentiate between materials based on the concentration of their spectral properties. Hyperspectral imaging combines spectroscopy with conventional imaging to collect both spatial and spectral information, producing a multidimensional hypercube. The camera captures 224 spectral bands within the NIR range of 942–1723 nm at a frame rate of 527 lines per second, with a spectral sensitivity of approximately 3.5 nm per band. This fine granularity allows the system to register subtle material differences. However, the first and the last 20 bands are less informative and noisier due to weaker signal strength. These bands were excluded in our procedure so as to improve data quality, leaving 184 spectral bands. The Specim FX17 camera has a spatial resolution of 640 pixels. In our setup, the camera was mounted orthogonally to the conveyor belt at a distance of 40 cm, measured from the lens to the belt. Positioned this way, the camera’s field of view covered a width of 300 mm, resulting in a pixel resolution of 0.47 mm. This resolution ensured that foreign objects with a minimum size of 1 mm^2^ would be captured by at least two pixels along the line scan, satisfying the detection requirements. Each acquired image comprises a hypercube of dimensions 640×1000×184.

The lighting system consisted of four commercial halogen bulbs, providing an intensity of 45,000 lux at the belt level. The lights were positioned on either side of the camera along the conveyor belt to ensure consistent illumination and avoid shadow formation on the imaging line.

### 2.3. Dataset Creation

**Figure 4:**
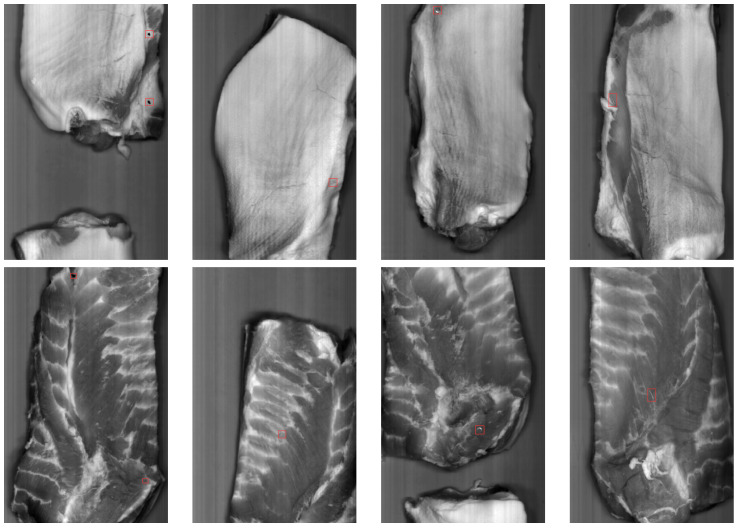
Examples of eight hyperspectral images: four with fat side up (**top row**) and four with meat side up (**bottom row**). Each image corresponds to one of the 184 spectral bands, acquired at wavelength 1046.5 nm, highlighting the presence of different contaminants.

In real-world production environments, contamination events are rare, making it impractical to capture a sufficient number of naturally occurring cases to train a robust detection model. To address this challenge while maintaining industrial relevance, we introduced a controlled dataset that incorporated a representative number of contaminants. Although realistic samples with a single foreign object were available from the production environment, as depicted in Figure 4, it was deemed more practical for training purposes to artificially introduce multiple contaminants into the dataset. This approach enabled effective system testing directly on the production line, accurately reflecting operational conditions while supporting rigorous model training and evaluation.

We collected 78 hyperspectral images of pork belly with contaminants and 183 images without contaminants, in order to generate the training and testing sets for the machine learning models. The images were captured under the same setup as described in Section 2.2. Both orientations of the pork belly—fat side up and meat side up—were considered, across various temperature ranges.

**Figure 5:**
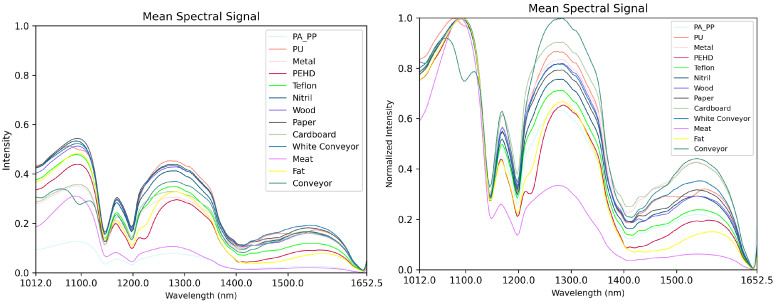
Hyperspectral mean intensity curve plots for our 13 test materials after flat-field correction, before (**left**) and after normalization (**right**).

From the 78 hyperspectral images of pork belly with contaminants, we manually annotated 22 images to train the segmentation model. The remaining 56 images were used for testing. Each annotated image was divided into 20×16 tiles with a 50% overlap for the tile-based segmentation approach, resulting in a total of around 80,000 tiles in the training set. It should be noted that the detection of fine contaminants was particularly challenging due to the surrounding material often influencing their spectral response. The mixed spectral signature between the contaminant and the surrounding material made it difficult to accurately determine the extent of the contaminant, leading to some imprecision in the annotated regions. Figure 5 presents the mean spectral signatures of the annotated materials.

**Figure 6:**
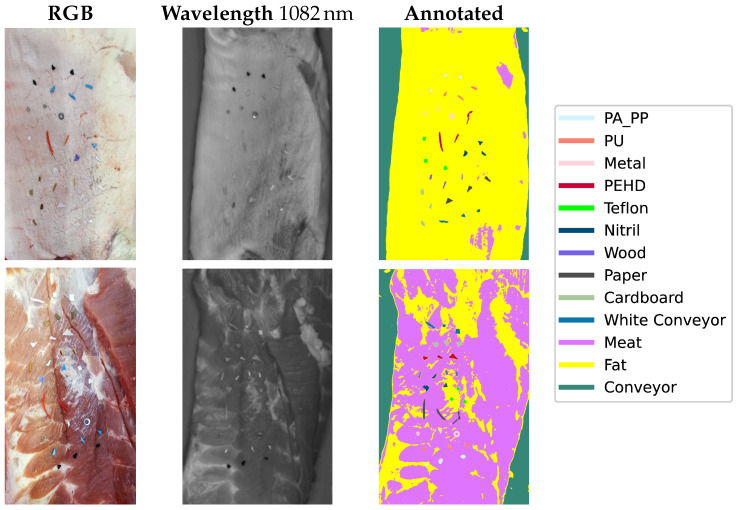
Examples of annotated data.

Figure 6, on the other hand, illustrates examples of the annotated training images. The first column depicts conventional colour images for better visualization, captured using a standard consumer-grade camera. The second column shows one of the 184 spectral frequency gray-level images that made up a hyperspectral cube, and the corresponding annotated segmentation masks in the third column. The last column introduces the colour associated with each material for annotation.

### 2.4. Input Conditioning and Normalization

Before feeding the hyperspectral images to the machine learning model, they were processed in two stages. The first stage involved flat-field correction to eliminate fixed-pattern vertical stripe noise, followed by pixel-wise normalization. These preprocessing steps ensured invariance under fluctuations in camera sensor temperature and exposure parameters.

Fixed pattern noise is temporally coherent and occurs due to pixel-to-pixel sensitivity variations in the sensor array, which are caused by factors such as differences in the cell response to incoming light, read-out and amplification electronics or thermal noise, making system calibration a challenge. Image stripes can also be caused by dirt or dust on the sensor. Flat field correction was applied to address these issues. The process involved capturing two frames: a dark one, acquired with the shutter closed, to measure the response of the detector in the absence of incident light, and a flat field image (gain frame) from a homogeneous surface to measure the response of each photo-diode to incoming light. The dark frame helped estimate dark currents for calibration, while the flat-field image allowed for the correction of variations in illumination and detector response. A Teflon tile was used as a homogeneous surface. After subtracting the dark field (one image or an average of several), each pixel should output the same black value. After flat-field correction, the image of a homogeneous surface captured under uniform illumination should appear flat. The flat-field correction process is described by the following equation [32]:

(1) $$C=\frac{(I-D)m}{F-D},$$

where *C* is the calibrated image, *I* is the acquired image to be calibrated, *D* is the dark frame, *F* is the flat field image and *m* is the average pixel value of the corrected flat field image F−D.

Both dark and gain frames must be acquired using the same ISO, exposure settings, and illumination conditions as the image being calibrated. Using *G* to denote gain, the equation for flat-field correction can be rewritten as:

(2) $$G=\frac{m}{F-D},$$

This simplifies the flat-field correction to:

(3) C=(I−D)G

The gain, *G*, measures how much the corrected flat field, F−D, deviates from its mean and from being flat.

**Figure 7:**
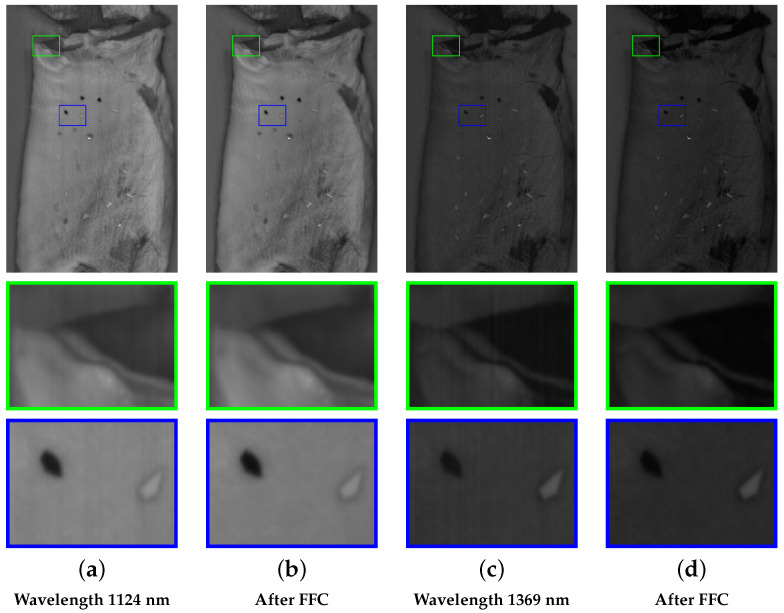
Flat-Field Correction eliminates vertical fixed pattern stripe noise.

Figure 7 illustrates an example of a hyperspectral image with visible fixed-pattern noise in columns (a,c), appearing as vertical stripes. Columns (b,d) depict the results after applying flat-field correction, with a reduced noise pattern. The crops highlight how the noise pattern varies across different wavelength images in columns (a,c).

**Figure 8:**
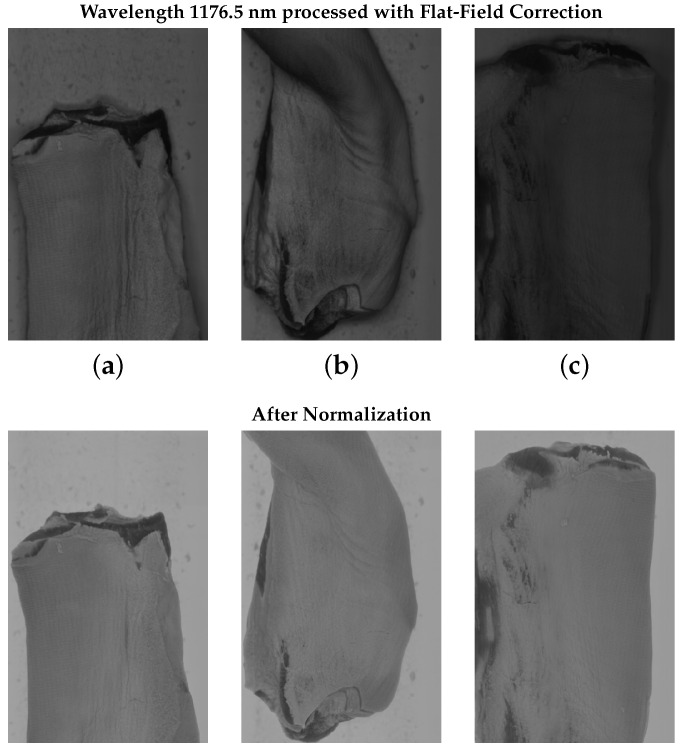
(**a**–**c**) Normalization produces similar hypercubes for hyperspectral images acquired under the same lighting conditions but different camera parameters and sensor temperatures. Normalization attenuates shadows, as seen in (**b**).

Figure 8, on the other hand, presents examples of slices from three hypercubes taken under the same illumination settings but at different camera temperatures. Columns (a,b) used the same camera exposure settings, while column (c) had a different exposure. In the top row, images captured at 1176.5 nm wavelength reveal significant differences in gray-level intensity between samples under different conditions. Shadows and variations in illumination also caused noticeable discrepancies. To address this, we applied pixel-wise normalization by subtracting the minimum spectral value across the 184 bands and then dividing by the maximum spectral value. The resulting normalized images are illustrated in the bottom row of Figure 8, demonstrating that after normalization, intra-class variability was reduced for fat, meat, and conveyor belt samples. Shadows and fine illumination differences were also attenuated, making the model more robust to variations in sensor temperature and exposure settings. Without normalization, there was a higher variability within each class, which would require more annotated samples to obtain better generalization. Without this step, distinguishing between samples of meat and fat became more challenging in the presence of shadows. Spectral normalization thus simplified the annotation task, reducing the need for extensive data labelling.

### 2.5. Segmentation Model

Despite the advancements referenced in Section 2.1, classification models still face challenges, as hyperspectral data often comprise hundreds of bands, with many materials exhibiting very similar spectral signatures. Moreover, existing methods predominantly adopt a tile-based approach, dividing hyperspectral images into small overlapping tiles and classifying only the center pixel of each tile. While this increases the number of training samples, it significantly prolongs training time due to the extensive overlap and computational overhead.

**Figure 9:**
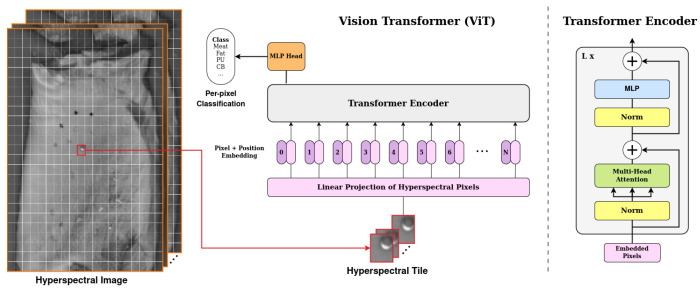
Overview of the ViT-based Architecture for HS Classification.

In contrast, our method classifies all pixels within each tile while leveraging neighbouring context, treating the task as dense segmentation rather than center-pixel classification. We divide the hyperspectral image into overlapping tiles of 20×16 pixels in size. Each tile is treated as an independent input to the network, allowing the model to leverage both spectral and spatial information. The architecture consists of a series of four cascaded transformer blocks, inspired by the Vision Transformer (ViT) introduced by Dosovitskiy et al. [33]. Each transformer block is composed of a self-attention module followed by a two-layer MLP, with each component preceded by a normalization layer and containing residual connections. The self-attention module consists of eight attention heads that, unlike the original ViT—which operates on non-overlapping image patches treated as tokens—computes self-attention across all pixels within a tile, progressively refining the learned spatial-spectral representations of the pixels. The embedding length remains fixed at 184 throughout the network, corresponding to the dimensionality of the hyperspectral pixel. This design choice enables the model to capture the aforementioned dependencies and interactions between neighbouring pixels, thereby improving classification accuracy. Finally, a fully connected linear layer is applied to the output of the last transformer block to classify each pixel, as depicted in Figure 9.

All our models were trained on an NVIDIA A100 Tensor Core GPU (Nvidia, Santa Clara, CA, USA) for 24 epochs with a batch size of 520. We utilized the AdamW optimizer with a weight decay of $2\times 10^{-4}$ and learning rate of $1\times 10^{-3}$, decayed using a polynomial learning rate scheduler with a warm-up of 3 epochs. A custom loss function was built on top of the standard cross-entropy loss to address the issue of false positives, particularly when meat or fat was misclassified as a contaminant. This loss function applied higher penalties for such misclassifications, reducing the false-positive rate, while assigning lower penalties when the conveyor belt was misclassified as a contaminant as this was not as critical. Additionally, label smoothing was incorporated with a rate of 0.3 to soften the hard labels, which helped mitigate the impact of imprecise annotations, especially at the boundaries of contaminants. The models were implemented in PyTorch 1.11.0 and Python 3.8.10. The source code with all hyper-parameter configurations and pre-trained models will be made available at https://github.com/hayatrajani/hsi-pork-vit (accessed on 13 November 2025).

All trained models were then evaluated on a standard laptop equipped with two NVIDIA GeForce GTX4060 GPU (Nvidia, Santa Clara, CA, USA) and an Intel Core i7-9700TE CPU (Intel, Santa Clara, CA, USA) operating at 3.80 GHz running Ubuntu 20.04.5, Python 3.8.10 and PyTorch 1.9.0+cu111. Model performance will be reported in terms of mean Intersection over Union (mIoU) and inference speed in the number of images processed per second (FPS).

### 2.6. Output Filtering

Given the industrial nature of this application, minimizing false positives was critical as each false alarm triggers manual inspection and increases costs. At the same time, missed contaminants are a safety risk. We therefore designed both the model and the loss to favour high specificity while retaining high detection accuracy as described in the previous section. However, a key challenge was imperfect ground truth at object borders. Annotations were reliable at the center of foreign objects, but boundary pixels were often ambiguous. We used label smoothing to reduce the penalty for near-border disagreements and to make the model less confident in uncertain regions; however, residual edge noise still remained, which we addressed with post-processing.

To suppress false positives, we applied morphological erosion to the detected contaminants with a 3×3 kernel as the structuring element. Although this step eliminated small blobs of false positives, but it also removed low-confidence predictions near the borders of contaminants, and excluded fine contaminants such as elongated or spiral-shaped PEHD with a width of 1–2 pixels. Contaminants without at least one 3×3 blob were reclassified as fat for such cases.

**Figure 10:**
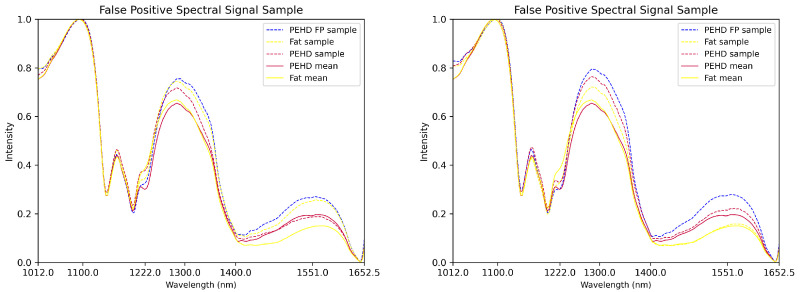

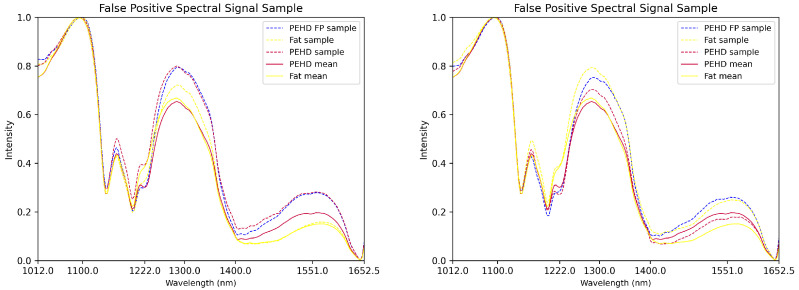
Plots of fat samples labeled as PEHD by the model with spectral curves that look more similar to PEHD samples than fat.

Furthermore, we noticed scenarios where the spectral signals of certain contaminants were very close to that of the background. Figure 10 illustrates cases where spectral signatures of PEHD false positives closely resembled those of true PEHD contaminants. Similarly, PEHD and fat sometimes exhibited extremely similar spectral curves, complicating their separation. This was similarly observed for PA-PP. Therefore, in addition to erosion, three pixel-wise post-processing conditions were applied to separate true from false positives based on their spectral characteristics, especially for PEHD and PA-PP:

1. If the model predicts that the current sample is PEHD but I(x,y,1225.5)−I(x,y,1211.5)>I(x,y,1026)−I(x,y,1012) and I(x,y,1411)/I(x,y,1407.5)<1.04) then that sample is labeled as fat.
2. If the model predicts that the current sample is PEHD but I(x,y,1117)>I(x,y,1099.5) then that sample is labeled as a conveyor belt.
3. If the model predicts that the current sample is PA-PP but I(x,y,1225.5)−I(x,y,1215)<I(x,y,1232.5)−I(x,y,1012) then that sample is labeled as meat, where I(x,y,w) denotes the gray-level intensity at the spectral wavelength *w* and pixel coordinates (x,y).

Together, the morphological filter removes small artifacts, and the spectral rules eliminate most remaining false positives in materials with spectra close to fat, while preserving high recall on true contaminants.

## 3. Results

**Figure 11:**
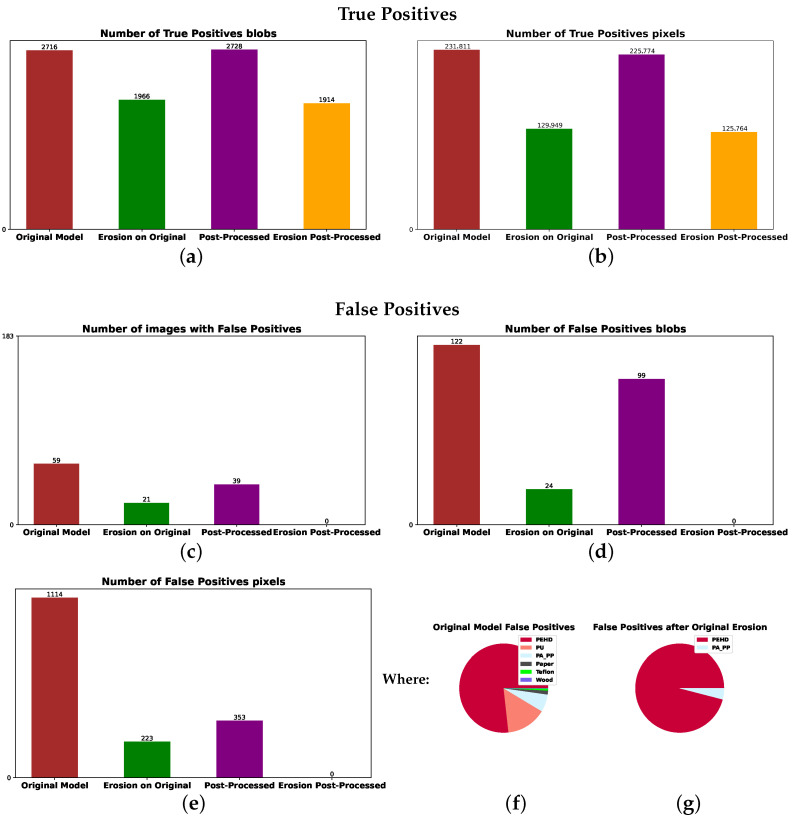
Prediction analysis of the results for the proposed model using the normalizing step, showing true positives as blob count in (**a**) and pixel count in (**b**), followed by false positives as image count in (**c**), blob count in (**d**) and pixel count in (**e**). The false-positive pixels counted in the brown bar of (**e**) have the material distribution from (**f**) before erosion, while those counted in the green bar of (**e**) have the material distribution from (**g**).

This section presents the results of two of our best-performing models: one incorporating spectral normalization as a preprocessing step and one without it. The results for the model with spectral normalization are shown in Figure 11. True-positive detections from the test set of 55 images with contaminants are presented in plots (a,b). For comparison purposes, the false positives from 183 images without contaminants are presented in plots (c–g). The brown bars represent results from the original model, and the green bars correspond to results obtained after performing morphological erosion. Purple bars show the results after applying the three post-processing conditions to the results of the original model, and orange bars represent the results after applying the three post-processing conditions followed by morphological erosion.

After applying morphological erosion, contaminant predictions were reduced to the most confident regions, primarily the interiors of blobs, leading to a significant drop in the number of predicted contaminant pixels for small objects. This effect is evident when comparing brown bars with green ones and purple bars with orange ones in plots (a,b) in Figure 11. Importantly, the orange bars of 0 height in plots (c–e) show that the three post-processing steps followed by erosion completely eliminated false positives from the test set while retaining most true positives, as seen by the minimal difference between orange and green bars in (a,b).

The false positives predicted by the original model are mostly PEHD, as seen in the pie plot from Figure 11f representing the distribution of the brown bar in (e). After the erosion step, there are still mostly PEHD false positives but also several PA-PP false positives, as seen in the (g) pie plot representing the distribution of the green bar in (e), motivating us to implement our three post-processing steps.

**Figure 12:**
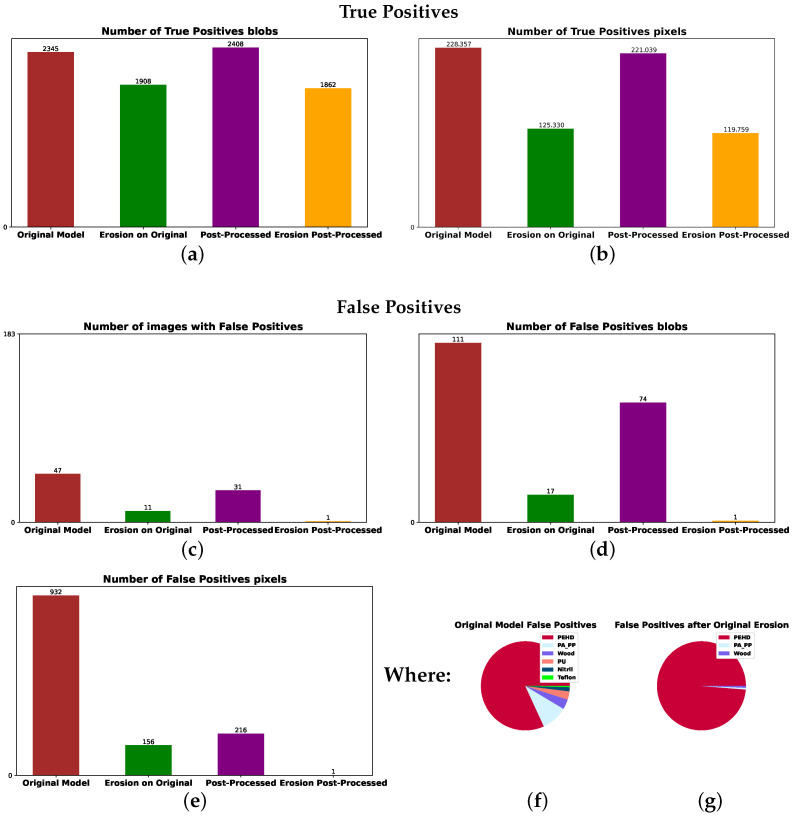
Prediction results for the model without normalizing, showing true positives as blob count in (**a**) and pixel count in (**b**), followed by false positives as image count in (**c**), blob count in (**d**) and pixel count in (**e**). The false-positive pixels counted in the brown bar of (**e**) have the material distribution from (**f**) before erosion, while those counted in the green bar of (**e**) have the material distribution from (**g**).

We also evaluated a model without the spectral normalization step, as depicted in Figure 12, using the same test set of 55 images with contaminants for evaluating true-positive detection as in plots (a,b) and 183 images without contaminants to estimate how many false positives appear, as in plots (c–e). The results confirmed the model with spectral normalization to be the most robust, followed by the post-processing and erosion steps. It achieved no false positives on the 183-image test set while retaining most true-positive predictions from the original model.

**Figure 13:**
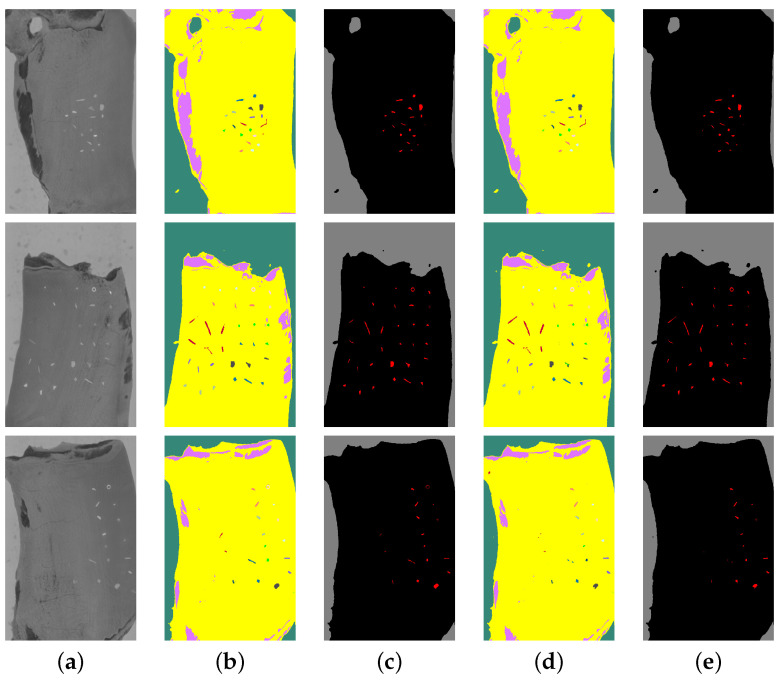
Prediction examples on fat. (**a**) Spectral wavelength 1169.5 nm; (**b**) prediction of the normalizing model; (**c**) prediction of the normalizing model followed by post-processing and contaminant erosion; (**d**) prediction of the model without the normalizing step; (**e**) prediction of the model without normalizing step followed by post-processing and contaminant erosion.

**Figure 14:**
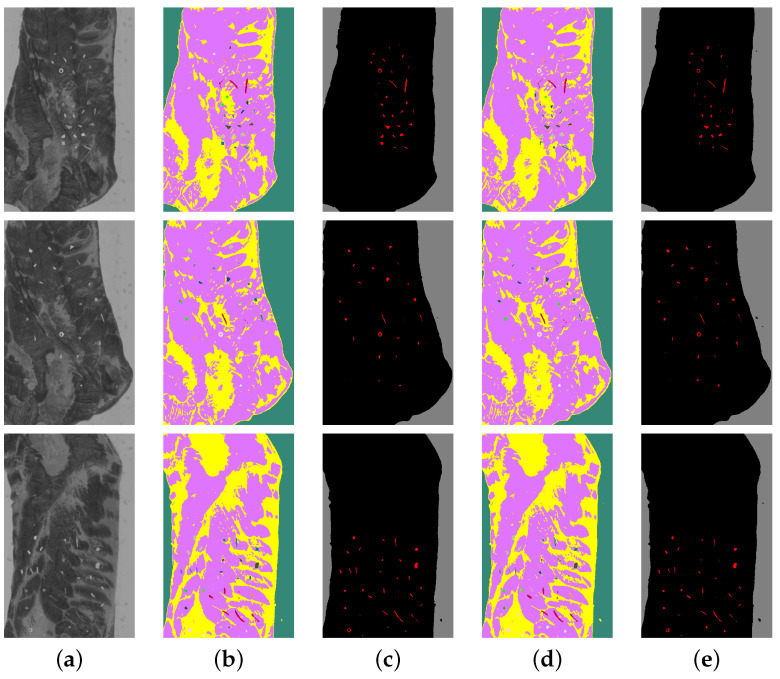
Prediction examples on meat. (**a**) Spectral wavelength 1169.5 nm; (**b**) Prediction of the normalizing model; (**c**) Prediction of the normalizing model followed by post-processing and contaminant erosion; (**d**) Prediction of the model without normalizing; (**e**) Prediction of the model without normalizing followed by post-processing and contaminant erosion.

Figure 13 and Figure 14 introduce several prediction examples from the test set with contaminants, showing a successful true-positive prediction for both fat and meat. In both figures, the prediction images in (b,d) have the same material colours as for the annotation in Figure 6, while the prediction masks in (c,e) use a simplified colour notation: red for samples of contaminant, black for pork and gray for conveyor belt.

**Figure 15:**
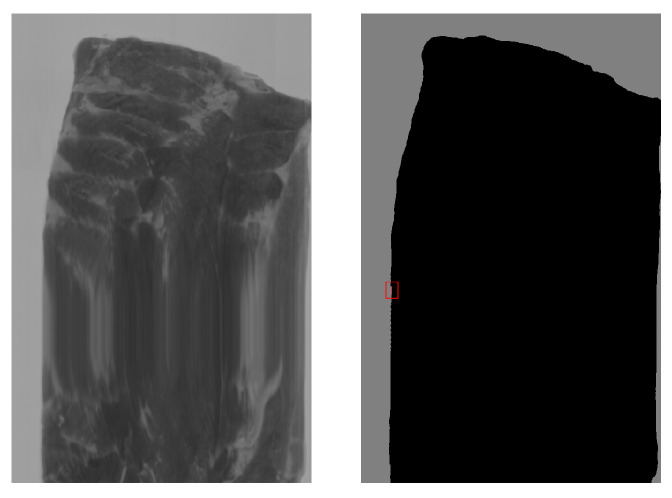
Image with false positives marked in red.

To further assess robustness, we tested the model with spectral normalization on an additional set of 208 hyperspectral images acquired under the same illumination and camera parameters but across a wide range of sensor temperatures (10–55 °C). This dataset presented typical variations in temperature encountered on an industrial production day. The model produced false positives in only one image, which included a 7-pixel blob labelled as nitrile and an 11-pixel blob labelled as white conveyor belt when the sensor temperature was 48 °C. This image, shown in Figure 15, was malformed, explaining the misclassification.

Finally, we evaluated the model on a challenging dataset of 95 hyperspectral images of pork belly on the conveyor belt containing blobs with spectral curves similar to the blue curves in Figure 10 around the inflection point at frequency 1222 nm. The model with spectral normalization, post-processing, and erosion steps produced no false positives on this dataset, further demonstrating its robustness in separating challenging contaminants like PEHD from other materials.

## 4. Discussion

In this study, we implemented a segmentation-based approach for detecting foreign objects on pork belly meat using hyperspectral imaging and a lightweight Vision Transformer. Unlike the methods in the literature that classify only the center pixel of overlapping tiles–resulting in significant computational overhead–, we densely classified all pixels within a tile while leveraging the spatial context of neighbouring pixels, which reduced training time and helped handle spectral similarity between contaminants and pork belly. In our experiments, this design, combined with normalization and light post-processing, achieved high detection accuracy with near-zero false positives under production-line variability.

It must be noted that we observed the temperature of the camera fluctuated significantly in real industrial scenarios, which affected flat-field/dark-frame corrections and, in turn, prediction quality. Dark frames and flat fields should therefore be computed for each sensor temperature value or range. Temperature fluctuations also influenced the average gray-level intensity and noise level, which was addressed by normalization. Although we chose a bright material like Teflon as the homogeneous surface for flat field correction, future work could include materials with different gray-level intensities and construct one model that all the information fuses in the flat field correction process.

For a model to be considered well-performing, we required reliable true-positive detection (contaminants correctly identified as contaminants) and no false positives (misclassification of meat, fat, or conveyor belt as contaminants). False positives matter operationally because they trigger manual inspection and unnecessary product loss, thereby increasing costs. Of the ten investigated contaminants, the fine pieces of PEHD contaminant are the most challenging to segment. The plot examples in Figure 10 illustrate the hardest case. During our numerous experiments testing different architectures, we found that all models pose difficulties when hyperspectral samples have similar curves to these examples, which motivated the post-processing strategies. This supported inline use where stable performance and low false-alarm rates were required. Additionally, ground truth was strongest at object centers and weaker at edges. Label smoothing reduced overconfidence near borders, but some edge noise remained. Post-processing removed most of this residual noise as well.

Nevertheless, these steps also eliminated some of the true positives. This mainly occurred at contaminant boundaries, increasing false negatives. For larger contaminants this was not a problem, as they were not completely eroded and retained a confident core. However, this remained a concern for very thin samples where the spectral signature at the boundaries of foreign bodies merges with that of surrounding material. This is a common diffraction effect in all image-formation processes that remains a challenge. The current spectral rules are simple and class-specific, and may need re-tuning for new products or lighting. Future work will replace hard spectral rules with a learned verifier or uncertainty-based filtering, guided by a complete sensitivity and specificity analysis across operating conditions.

## Data Availability

The original data presented in the study are openly available in Zenodo at https://zenodo.org/records/17242553 (accessed on 1 October 2025).
